# The crosstalk of breast cancer and ischemic heart disease

**DOI:** 10.1038/s41420-025-02428-6

**Published:** 2025-04-18

**Authors:** Yunbo Luo, Jun Liu, Peng Qu, Shiqi Han, Xue Li, Yali Wang, Xiaohan Su, Jiao Zeng, Jinsui Li, Shishan Deng, Qi Liang, Lingmi Hou, Panke Cheng

**Affiliations:** 1 Department of Breast Surgery, Sichuan Clinical Research Center for Cancer, Sichuan Cancer Hospital & Institute, Sichuan Cancer Center, Affiliated Cancer Hospital of University of Electronic Science and Technology of China, Chengdu, 610041 China; 2 Department of Academician (expert) Workstation, Biological Targeting Laboratory of Breast Cancer, Breast and Thyroid Surgery, Affiliated Hospital of North Sichuan Medical College, Nanchong, Sichuan 637000 P. R. China; 3 Institute of Cardiovascular Diseases & Department of Cardiology, Sichuan Provincial People’s Hospital, School of Medicine, University of Electronic Science and Technology of China, Chengdu, 610072 P.R. China; 4 Department of Clinical Laboratory, Affiliated Hospital of North Sichuan Medical College, Nanchong, 637000 People’s Republic of China; 5 School of Laboratory Medicine, North Sichuan Medical College, Nanchong, 637007 People’s Republic of China; 6 Translational Medicine Research Center, North Sichuan Medical College, Nanchong, 637007 People’s Republic of China; 7 Ultrasound in Cardiac Electrophysiology and Biomechanics Key Laboratory of Sichuan Province, Chengdu, 610072 P.R. China

**Keywords:** Breast cancer, Myocardial infarction

## Abstract

In recent years, the continuous optimization of anti-tumor therapy has greatly improved the cancer-specific survival rate for patients with breast cancer (BC). The prevention and treatment of breast cancer-related heart diseases have become a new breakthrough in improving the long-term survival for BC patient. The cardiac damages caused by BC treatment are increasingly prominent among BC patients, of which ischemic heart disease (IHD) is the most prominent. Besides, the systemic inflammatory response activated by tumor microenvironment c an induce and exacerbate IHD and increase the risk of myocardial infarction (MI). Conversely, IHD can also exert detrimental effects on tumors. MI not only increases the risk of BC, but also induces specialized immune cell to BC and accelerates the progression of BC. Meanwhile, the treatment of IHD can also promote BC metastasis and transition to more aggressive phenotypes. Although BC and IHD are diseases of two independent systems, their crosstalk increases the difficulty of anti-cancer treatment and IHD management, which reduces the survival for both diseases. Therefore, this review mainly explores the mutual influence and underlying mechanisms between BC and IHD, aiming to provide insights for improving the long-term survival for patients with BC or IHD.

## Facts

- Ischemic heart disease not only increases the risk of breast cancer but also induces immune cell activity that accelerates the progression of breast cancer.
- The systemic inflammatory response activated by the tumor microenvironment can exacerbate ischemic heart disease, increase the risk of myocardial infarction.
- The crosstalk between breast cancer and ischemic heart disease increases the difficulty of managing both conditions simultaneously, as treatments for one can impact the progression of the other, ultimately reducing survival for both diseases.
- Understanding the mutual influence and underlying mechanisms between breast cancer and ischemic heart disease can provide insights into improving treatment strategies and enhancing the long-term survival of patients with either or both conditions.

## Open questions

- What are the exact ways in which ischemic heart disease influences tumor progression, immune cell recruitment, and metastasis in breast cancer, and how can this knowledge be used to develop therapies that prevent or reduce this effect?
- What novel strategies can be developed to improve the long-term survival of patients with both breast cancer and ischemic heart disease, considering their complex interplay and mutual influence?
- What are the best approaches for managing both breast cancer and ischemic heart disease simultaneously, considering the interactions between treatments for both conditions?

## Introduction

Breast cancer (BC) is the most common malignant tumor among women and poses a serious threat to women’s physical health and life safety [1]. Fortunately, significant progress has been made in the diagnosis and treatment of BC. With the popularization of breast screening, an increasing number of patients with BC are being diagnosed at early-stage [2]. In addition, over the past few decades, comprehensive treatments for BC have been continuously improved, including surgery, radiotherapy, chemotherapy, targeted therapy, hormone therapy, and immunotherapy. The surgical approach for BC has evolved from traditional radical mastectomy and extended radical mastectomy to surgical techniques that also consider the aesthetic outcomes for women [3], such as breast-conserving surgery and breast reconstruction surgery. In addition to surgery, radiotherapy is also an important reinforce to the local treatment of BC, significantly reducing the risk of recurrence after breast-conserving surgery and mastectomy [4, 5]. Chemotherapy remains the primary systemic treatment for BC, aiming to shrink tumors and control the disease by killing tumor cells in the primary site or potential metastatic lesions. The advent of anti-HER-2 targeted therapy has been more than 20 years [6], from trastuzumab alone to trastuzumab in combination with pertuzumab [7]. In addition, other small molecule drugs targeting to HER2, such as lapatinib and neratinib, have also improve the long-term survival for patients with HER2-positive (HER2 + ) BC [8–10]. In patients with hormone receptor-positive (HR + ) BC patients, traditional endocrine therapy has already improved the long-term survival of this population, including basic endocrine drugs such as tamoxifen, aromatase inhibitors, fulvestrant, and etc [11–13]. In recent years, CDK4/6 inhibitors have further improved the prognosis for patients with high-risk of recurrence in hormone receptor-positive BC [14], and it has also significantly improved the progression-free survival and overall survival for patients with advanced BC [15]. Immunotherapy also has some effect in the treatment of BC, and its combination with other treatment regimens has shown promising outcome in triple-negative breast cancer (TNBC) [16, 17]. Apart from these treatment approaches, a multitude of promising therapeutic drugs continue to emerge on the clinical horizon. Such as sacituzumab govitecan, a promising drug targeting Trop-2, due to its outstanding effect for patients with HR + /HER2- advanced BC [18], has been approved by the Food and Drug Administration (FDA) for unresectable locally advanced BC or metastatic HR + /HER2- BC with prior endocrine therapy and ≥2 lines of systemic therapy.

Because of these advances in comprehensive treatment, the outcome of BC has been greatly improved. A recent study from Stanford University School of Medicine showed that the mortality rate from BC in the United States decreased by 58% absolutely in 2019 compared with 1975, especially a 71% decrease in ER + /HER2 + BC [19]. A database analysis showed that the long-term survival rate of BC patients has also improved significantly in Northern European countries, which is close to the higher survival rate of BC patients in Sweden (with 5-year and 10-year survival rates of 92.3% and 87.8%, respectively) [20]. In China, a retrospective study showed that the 5-year and 10-year overall survival rates of patients with operable BC were 92.9% and 87.4%, respectively [21]. A recent statistical analysis in Korea also showed that the 5-year survival rate of BC is as high as 93.6% [22]. Thus, in the context of relatively complete comprehensive treatment for BC, the specific survival time of BC will be further prolonged, and more and more patients will die from non-breast cancer disease, such as cardiovascular disease, respiratory system disease, chronic liver disease, suicide, and etc [23–25]. An analysis of Surveillance, Epidemiology, and End Results (SEER) database showed that cardiovascular disease was the leading cause of death in elderly women with BC (15.9% (95% CI 15.6 to 16.2)), followed by BC (15.1% (95% CI 14.8 to 15.4)) [26]. Then, another large cohort study also showed that as patients with early-stage BC survived longer, cardiovascular disease had surpassed BC and become the leading cause of death in patients with early-stage BC [27]. Previous studies have confirmed that BC survivors are more likely to develop cardiovascular disease [28, 29], which may be mainly caused by multiple anti-cancer regimes [30]. Dyslipidemia is not only a common side effect of anti-cancer treatments, such as endocrine therapy and chemotherapy, but also the main cause of atherosclerotic cardiovascular disease [31, 32]. Therefore, abnormal lipid metabolism may contribute to breast cancer-related cardiovascular disease in patients with BC. In addition, a variety of chemotherapeutic drugs can cause cardiovascular damage. Anthracyclines are commonly used chemotherapeutic drugs for BC, which can cause irreversible cardiac damage through various mechanisms, such as oxidative stress, changes in cell death pathways and epigenetic alterations [33, 34]. Anti-HER2 targeted therapies, such as trastuzumab, can also cause reversible cardiac dysfunction [35], which will further increase cardiotoxicity when combining with anthracycline [36]. In recent years, the use of CDK4/6 inhibitors can also increase the risk of venous thrombosis in cancer patients [37, 38]. Immune checkpoint inhibitors also have some cardiovascular toxicity [39]. Postoperative radiotherapy can also promote myocardial ischemia and myocardial infarction (MI) in BC patients [40, 41]. Thus, breast cancer-related cardiovascular disease will become the main cause of death for patients with BC.

Cardiac injury is the most common type of cardiovascular disease caused by tumors and their treatments, which has attracted more and more attention from oncologists and cardiologists. It manifests as related symptoms such as heart failure, arrhythmia, myocarditis, pericarditis, pericardial effusion and ischemic heart disease (IHD) [42]. IHD often occurs in patients with malignant tumors, especially in BC survivors. In a study with median follow-up of 8 years, 986 out of 13,348 BC patients experienced cardiovascular events, including 736 (74.6%) with coronary heart disease [43]. At the same time, ischemic heart injury develops rapidly and is dangerous, which brings certain difficulties to diagnosis and treatment. Once MI occurs, it is often difficult to reverse and the disability rate and mortality rate are extremely high [44, 45], which poses a serious threat to the long-term survival for BC patients. Therefore, it is crucial to clarify the crosstalk between BC and IHD for improving long-term survival of patients with BC.

## Interaction between BC and IHD

### Impact of BC on IHD

**Fig. 1:**
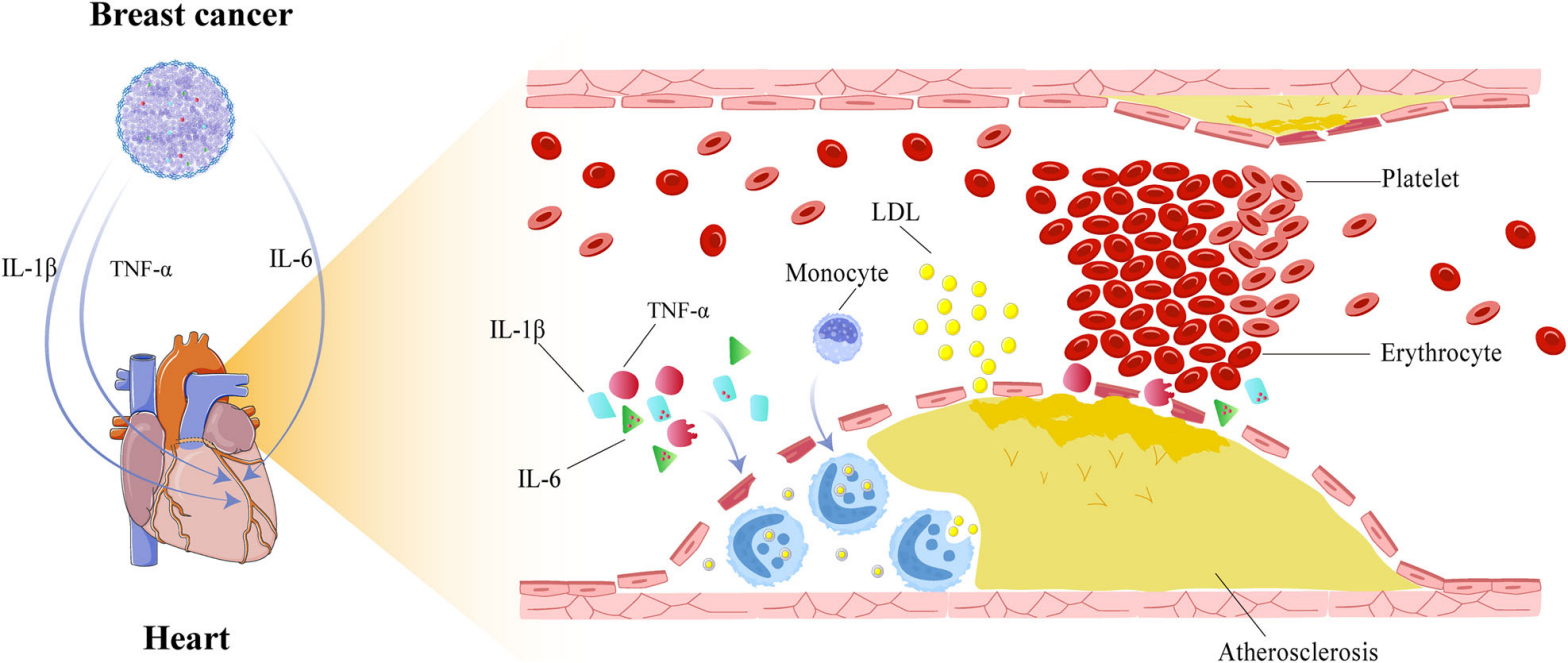
The breast cancer accelerates ischemic heart disease. The inflammatory factors occurring in the microenvironment of breast tumor injury the vascular endothelial cells, which contributes to ischemic heart disease by inducing the formation of atherosclerosis and thrombosis. IL-1β, interleukin-1 beta；TNF-α, tumor necrosis factor-alpha；IL-6, interleukin-6；LDL, low density lipoprotein.

There are many common risk factors for tumors and heart diseases, such as obesity, smoking, inflammation, etc [46]. Nevertheless, previous studies have shown that patients with BC are more likely to develop myocardial ischemia (hazard rations [HR] = 1.8, 95% confidence intervals [CI]: 1.0–3.1) and MI (HR = 2.9, 95% CI: 1.9–4.2) than those without BC [47]. Therefore, in addition to the shared risk factors for BC and IHD, BC may also precipitate ischemic changes in the heart, exacerbating the incidence of MI. The occurrence of tumors can induce a series of immune responses in the body, leading to the production of various pro-inflammatory cytokines, such as tumor necrosis factor-alpha (TNF-α), interleukin-1 beta (IL-1β), interleukin-6 (IL-6), transforming growth factor beta (TGF-β), etc [48, 49]. These cytokines can also inflict damage on vascular endothelial cells, potentially exerting more pronounced effects on the coronary arteries and their branches because of rich blood supply. This could accelerate the IHD in patients with BC (Fig. 1 and Table 1).

**Table 1:** Interaction between BC and IHD.

Impact of BC on IHD	Pro-inflammatory cytokines facilitate the progression of IHD.	TNF-α	Damage vascular endothelial cells. Stimulate inflammatory responses. Reducing nitric oxide bioavailability.
		IL-1β	Induce a robust inflammatory cascade. Stimulate the expression of adhesion molecules. Upregulating the expression of P2Y2.
		IL-6	Induce the production of acute-phase proteins. Stimulate vascular smooth muscle cell proliferation and migration. Weaken the fibrous cap of plaques.
		TGF-β	Promote cardiac fibrosis. Maladaptive myocardial remodeling. TGF-β signaling contributes to inflammation.
	Tumor-derived metabolites exacerbate the progression of IHD.	Heightened glucose demand	Exacerbate metabolic stress on the myocardium and elevate the risk of cardiac hypoxia.
		Fatty acids consumption	Impact the heart’s fatty acid metabolism.
The Impact of IHD on BC	Changes after MI affect BC.	Extracellular vesicles	Specific biomolecules promote the progression of BC.
		Immune cell reprogramming	Cause systemic immune dysregulation and accelerate the progression and metastasis of BC.
		MI induced heart failure	NGF-TRKA signaling pathway promotes BC growth.
	Metabolite changes promote BC.	Anaerobic glycolysis	Acid accumulation promotes tumor proliferation and metastasis.

*BC* breast cancer, *IHD* ischemic heart disease, *MI* myocardial infarction, *TNF-α* tumor necrosis factor-alpha, *IL-1β* interleukin-1 beta, *IL-6* interleukin-6, *TGF-β* transforming growth factor beta.

TNF-α is primarily produced by activated macrophages and tumor cells, with contributions from fibroblasts, T lymphocytes, and natural killer cells, promoting its accumulation within the tumor microenvironment. Early studies have confirmed that TNF-α expression is significantly higher in BC tissues compared to normal breast tissues, and it is mainly produced by stromal cells [50]. It can promote the proliferation and distant metastasis of breast cancer cells [51, 52]. Subsequent studies have shown that serum TNF-α levels in BC patients are positively correlated with tumor size, TNM stage, and lymph nodes metastasis [53, 54]. Apart from its involvement in tumor invasion and metastasis, TNF-α plays a crucial role in the occurrence and progression of coronary heart disease. Previous research has shown that TNF-α can directly damage vascular endothelial cells, leading to increased vascular endothelial permeability, thereby promoting lipid deposition within the vessel wall and causing the formation of coronary atherosclerotic plaques [55]. Additionally, TNF-α can also stimulate inflammatory responses and induce changes, such as thrombus formation, vasoconstriction, and abnormal proliferation of vascular smooth muscle cells, exacerbating the risk of IHD and MI [56]. Furthermore, TNF-α can impair endothelial cell function by reducing nitric oxide (NO) bioavailability, leading to vascular dysfunction, reduced coronary blood flow, and increased risk of ischemia [57].

IL-1β, as an endogenous mediator of leukocytes, is an effective pro-inflammatory cytokine involved in the regulation of autoimmune inflammation [58]. Elevated expression of IL-1β can be observed in the immunological microenvironment of various solid tumors, primarily produced by immune cells, fibroblasts, and tumor cells [59]. The elevation of IL-1β in the bloodstream of breast cancer patients was observed by previous study [60], and its expression is closely associated with adverse prognosis in BC [61]. In luminal BC, IL-1β promotes the production of IL-6 through the NF-κB signaling pathway, which leads to tumor growth and increased malignancy [62]. In cardiovascular diseases, IL-1β plays a critical role in mediating inflammatory responses in vascular endothelial cells. It induces a robust inflammatory cascade by activating endothelial cells and upregulating the production of pro-inflammatory cytokines and chemokines [63]. Additionally, IL-1β stimulates the expression of adhesion molecules, such as ICAM-1, VCAM-1, and E-selectin, on the surface of vascular endothelial cells [64]. These adhesion molecules are essential for recruiting circulating leukocytes, facilitating their rolling, firm adhesion, and eventual transmigration to sites of inflammation. The enhanced interaction between leukocytes and endothelial cells at inflamed vascular sites exacerbates vascular damage, promotes endothelial dysfunction, and contributes to the progression of cardiovascular diseases, including atherosclerosis and ischemia-reperfusion injury. Furthermore, it can accelerate the progression of atherosclerosis by upregulating the expression of P2Y2, a purinergic receptor that plays a significant role in vascular biology [65]. This upregulation promotes the proliferation and migration of vascular smooth muscle cells (VSMCs), which are key processes in the development and progression of atherosclerotic plaques. The enhanced activity of VSMCs contributes to the thickening of the vascular wall, the formation of neointima, and the destabilization of plaques, ultimately increasing the risk of vascular obstruction and cardiovascular events associated with advanced atherosclerosis.

IL-6 is a multifunctional cytokine and adipokine with dual roles in promoting both tumorigenesis and inflammation [66, 67]. It is primarily produced by non-tumor cells, such as monocytes, macrophages, T cells, B cells, fibroblasts, endothelial cells, and adipocytes [66, 68]. IL-6 can also be produced in BC cells [69], and high serum concentrations of IL-6 are closely associated with poor prognosis in BC [70]. In addition to the key role in the initiation and progression of tumors, IL-6 is also a typical cytokine involved in systemic inflammatory responses in the body and participates in the process of atherosclerosis. Gene data analysis indicates that the IL-6 signaling pathway is involved in various cardiovascular events associated with atherosclerotic lesions, such as MI and peripheral arterial disease [71]. Basic research has also shown that giving high-fat diet-fed mice with IL-6 significantly exacerbated the progression of atherosclerotic lesions in mice [72]. A randomized, double-blind, phase II clinical trial demonstrated that a monoclonal antibody targeting the IL-6 receptor (ziltivekimab) significantly reduced the biomarkers of atherosclerosis-related inflammation and thrombosis formation [73], which suggests that it could be helpful to reduce the risk of cardiovascular events by inhibiting the pro-inflammatory effects of IL-6. Therefore, high concentrations of IL-6 in BC patients may promote the occurrence of coronary atherosclerosis, myocardial ischemia, and MI. The mechanisms by which IL-6 contributes to the development of IHD can be elucidated through the following aspects. First, IL-6 can induce the production of acute-phase proteins such as C-reactive protein (CRP), which enhances monocyte adhesion to endothelial cells and accelerates atherosclerotic plaque formation [74]. Additionally, IL-6 stimulates vascular smooth muscle cell proliferation and migration, contributing to neointima formation and plaque growth [75]. Furthermore, IL-6 can weaken the fibrous cap of plaques by increasing matrix metalloproteinase (MMP) activity, making them more prone to rupture, leading to acute ischemic events [76].

BC cells can produce high levels of TGF-β, which plays a critical role in tumor progression, metastasis, and immune evasion [49]. In breast cancer, TGF-β contributes to epithelial-to-mesenchymal transition (EMT), enhancing the invasive and migratory properties of cancer cells, thereby promoting metastasis [77]. Additionally, TGF-β can suppress anti-tumor immune responses, further aiding tumor survival and progression [78]. Beyond its tumorigenic effects, elevated TGF-β levels in BC patients can have detrimental impacts on cardiovascular health, particularly in the context of IHD. TGF-β has been shown to promote cardiac fibrosis by inducing fibroblast activation and the deposition of extracellular matrix proteins in response to ischemic injury [79]. Chronic activation of TGF-β in the heart can lead to maladaptive myocardial remodeling, increasing the risk of heart failure [80]. Moreover, TGF-β signaling contributes to inflammation, which can exacerbate ischemic damage in the heart. This intersection between breast cancer and cardiovascular disease highlights the complex role of TGF-β in both tumor progression and heart tissue damage, making it a potential therapeutic target for modulating tumor growth while also addressing cardiovascular complications. Thus, targeting TGF-β may offer a dual benefit in managing both cancer progression and IHD.

In addition to pro-inflammatory cytokines, other factors may also contribute to the progression of IHD. The heightened glucose demand of tumor cells may competitively diminish the glucose availability for cardiac cells [81], particularly in individuals with IHD, thereby exacerbating metabolic stress on the myocardium and elevating the risk of cardiac hypoxia. Then, tumor cells require large amounts of fatty acids during their growth [82], which may alter the distribution of fatty acids in the bloodstream and impact the heart’s fatty acid metabolism. This, in turn, exacerbates metabolic disturbances during cardiac ischemia, particularly in conditions of hypoxia or reduced blood flow. Furthermore, tumor cells tend to produce lactic acid through glycolysis [83], and lactic acid accumulation can aggravate ischemic heart injury.

### The Impact of IHD on BC

The pumping function of the heart is crucial for normal physiological activities in the human body, and its ischemic alterations are also closely associated with the occurrence and progression of BC. A prospective study has shown a significant increase the incidence of malignant tumors in patients following heart failure post-myocardial infarction, especially BC in women [84]. Another study also demonstrated that the patients with MI has a 46% increase in the incidence of malignant tumors (HR = 1.46; 95% CI: 1.21–1.77) compared to the population without MI [85]. These studies suggest that ischemic alterations in the heart may increase the risk of developing breast cancer. Some scholars argue that IHD and BC share the common risk factors, such as reduced physical activity ( < 150 min/week) [86]. Obesity and overweight are primary risk factors for cardiovascular diseases [87], which are also risk factors for breast cancer in postmenopausal women [88]. A Mendelian randomization study demonstrated that smoking significantly increased the risk of coronary artery disease and MI [89], meta-analysis also indicated that both active and passive smoking will increase the risk of breast cancer [90]. Therefore, IHD is closely associated with the occurrence of BC, but further researches are needed to confirm that and clarify the potential mechanisms.

**Fig. 2:**
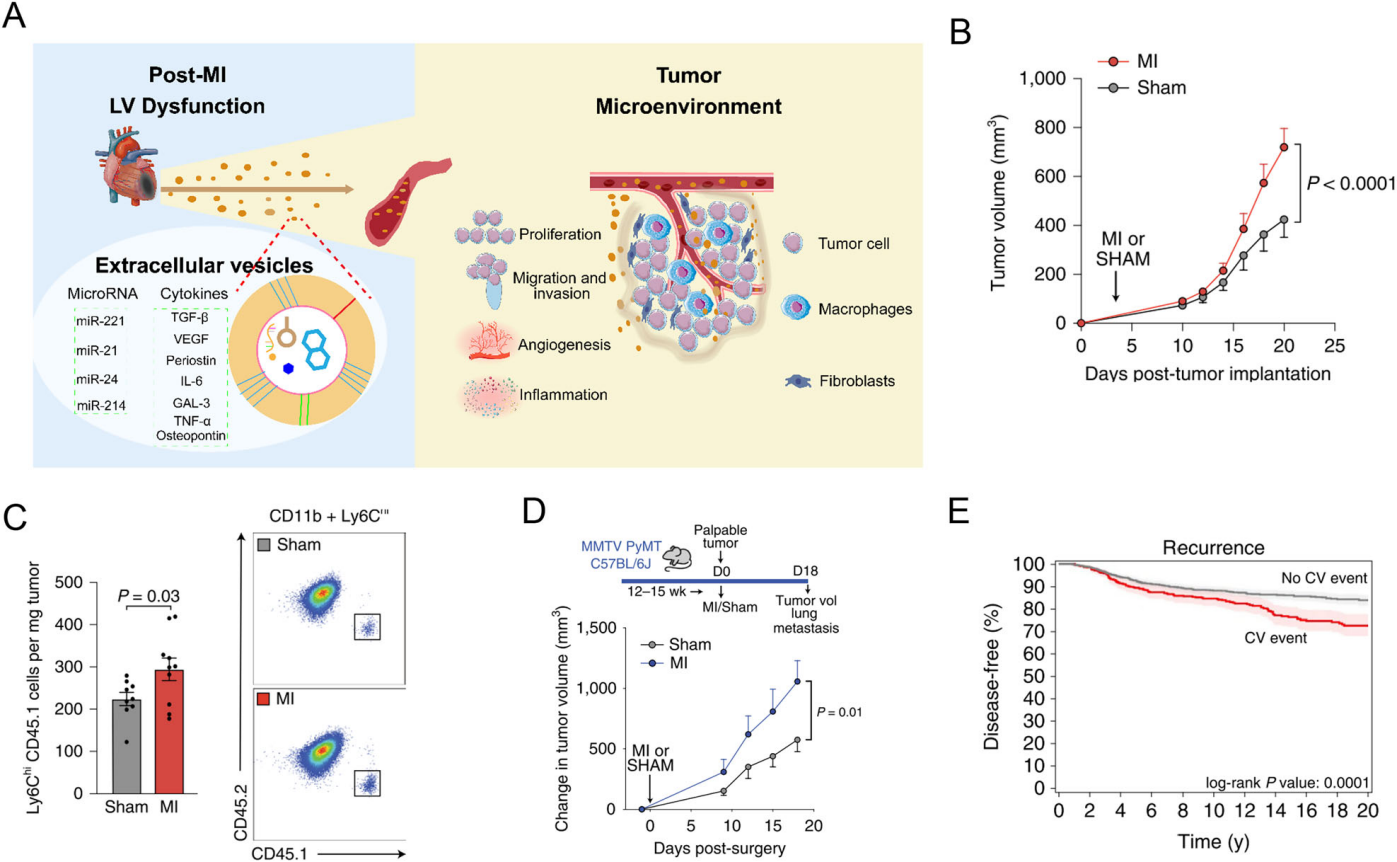
The ischemic heart disease promotes the progression and recurrence of breast cancer. **A** Cardiac interstitial stromal cells release extracellular vesicles containing specific biomolecules with reparative effects on the heart, such as osteonectin, IL-6, galectin-3, TNF-α, VEGF, etc. Extracellular vesicles enriched with these biomolecules promote tumor proliferation and migration upon reaching tumor tissue via the bloodstream [91]. **B** Myocardial infarction promotes the growth of transplanted breast cancer in mice; **C** Myocardial infarction promotes inhibitory immune cell production and aggregation in breast cancer; **D** Myocardial infarction promotes the growth and metastasis of spontaneous breast cancer in mice; **E** Cardiovascular disease accelerates breast cancer recurrence. Panels B-E were reproduced from the study by Koelwyn GJ et al, Nat Med. 2020 [92]. Copyright 2020, Springer Nature.

IHD not only increases the risk of BC but also promotes the progression and recurrence of BC (Table 1). Recently, a study conducted by Tel Aviv University confirmed the tumor-promoting effects of MI (Fig. 2A) [91]. After MI, cardiac interstitial stromal cells will release extracellular vesicles containing specific biomolecules with reparative effects on the heart, such as osteonectin, IL-6, galectin-3, TNF-α, VEGF, etc. Those specific biomolecules will promote the progression of BC. Furthermore, a study conducted by the New York University suggested that MI led to immune cell reprogramming, inducing some immune cells to adopt an immunosuppressive phenotype, thereby causing systemic immune dysregulation and accelerating the progression and metastasis of BC (Fig. 2B–D) [92]. Additionally, the team continued to retrospectively analyze two prospective cohorts of early-stage BC and found that those who experienced cardiovascular events (such as MI or stroke) after diagnosis of BC had a 59% increased risk of BC recurrence (Fig. 2E) (HR = 1.59, 95%CI: 1.23-2.05) and a 60% increased risk of BC mortality (HR = 1.6, 95%CI: 1.15-2.22) [93, 94]. Another study showed that insulin-like peptide 6 (INSL6) is downregulated in patients with MI, while BC patients with low INSL6 expression exhibited poorer overall survival. This suggested that MI may promote BC progression by downregulating INSL6 expression [95]. Moreover, a recent study suggests that MI can promote breast tumor growth through the NGF-TRKA signaling pathway [96]. IHD can induce structural changes in the heart and lead to heart failure, both of which have been shown to promote tumor progression in several preclinical studies. Early or mild heart remodeling can promote both tumor growth and metastasis [97, 98], and heart hypertrophy may release secretory factors that contribute to cancer development and progression [99]. Also, cytokines and growth factors secreted after heart failure can promote tumor progression through integrin receptors [100]. Besides, metabolic changes in IHD may also promote tumor progression. Anaerobic glycolysis is a primary metabolic pathway in IHD, leading to increased lactic acid accumulation, which in turn promotes tumor proliferation and metastasis [101, 102]. Therefore, IHD promotes the deterioration of BC, adversely affecting the prognosis of BC.

From the above, it can be inferred that BC and IHD are not two independent diseases; rather, they interact with each other. The occurrence of BC may trigger and accelerate IHD by activating the systemic inflammatory response system. Conversely, IHD can also promote the occurrence and progression of BC, significantly increasing the risk of BC recurrence.

## The crosstalk between BC treatment and IHD management

**Fig. 3:**
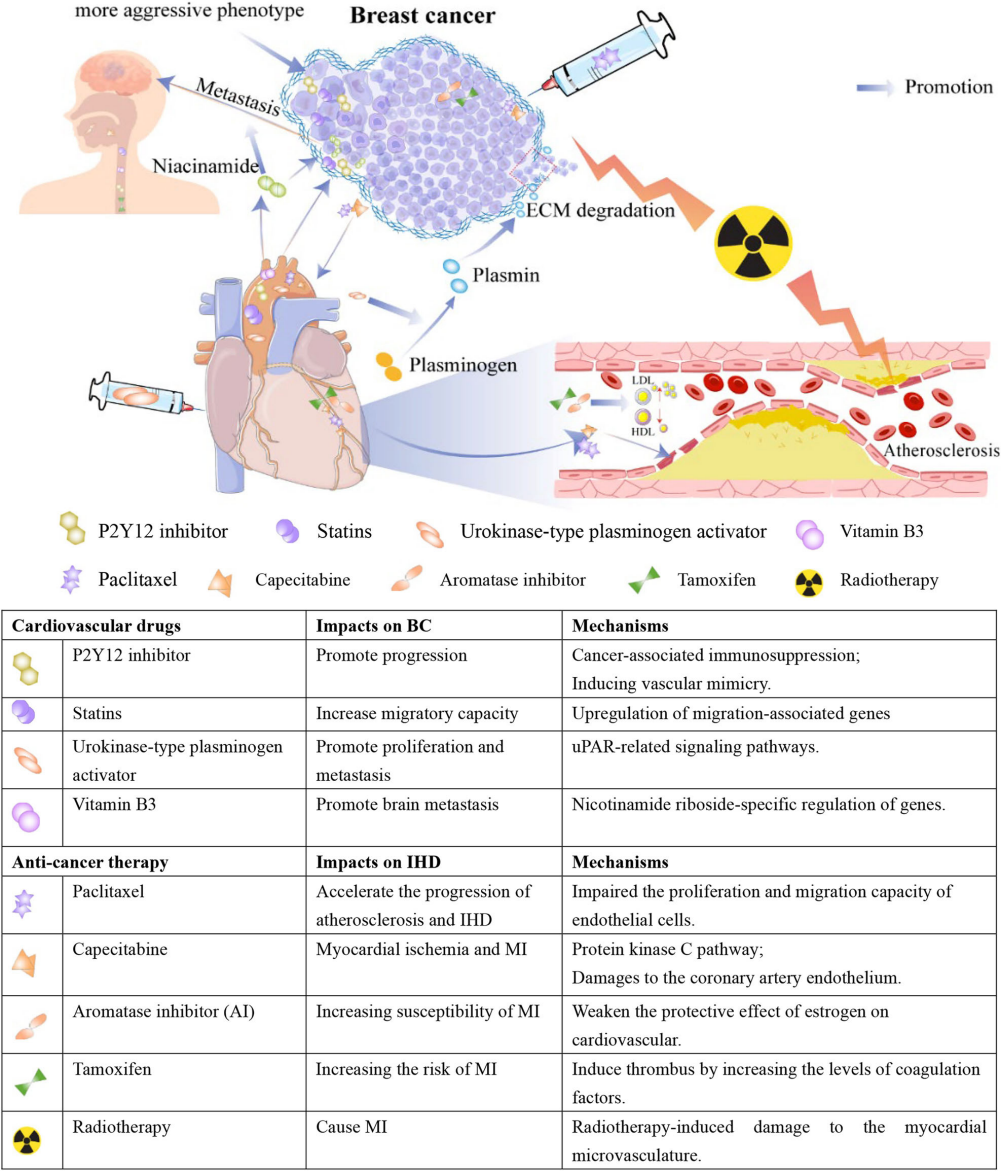
The crosstalk between breast cancer treatment and ischemic heart disease management. The treatment of breast cancer, such as chemotherapy and endocrine therapy and radiotherapy, lead to the ischemic heart disease by inducing the atherosclerosis and vasospasm. On the contrary, the management of ischemic heart disease, such as statins, P2Y12 inhibitor, urokinase-type plasminogen activator and vitamin B3, also promote the deterioration and metastasis of breast cancer.

In addition to their mutual influence on each other, the treatments for breast cancer and ischemic heart disease can also exert reciprocal effects. Many therapeutic regimens of BC, such as chemotherapy, endocrine therapy, and radiotherapy, may facilitate and accelerate the progression of ischemic heart disease. Conversely, many treatments for IHD, such as thrombolytic therapy and anticoagulant therapy, may also have a promoting effect on the occurrence and progression of breast cancer (Fig. 3).

### The impact of BC treatment on IHD

Most patients with BC require comprehensive treatment, including surgery, radiotherapy, chemotherapy, targeted therapy, and hormonal therapy. However, many of these treatments carry potential risks for cardiac damage. Postoperative radiotherapy for BC significantly improves the local control of breast cancer [5]; while, the damage to the heart caused by radiotherapy should not be ignored. A linear relationship was observed between the incidence of MI and the average total radiation dose to the heart in postoperative radiotherapy for MI [41]. Especially, postoperative radiotherapy in patients with left-sided breast cancer is more prone to induce MI [103]. These injuries may stem from radiotherapy-induced damage to the myocardial microvasculature and its promotion of atherosclerosis in larger arteries [41]. However, radiotherapy remains a necessary treatment modality for the majority of BC. Thus, it may help reduce cardiac damage by new radiotherapy techniques, such as intensity-modulated radiotherapy (IMRT), optimized radiotherapy plans, and reducing the radiation dose to the heart

Chemotherapy is an indispensable treatment modality for many breast cancer patients, and it plays a crucial role in neoadjuvant and adjuvant therapy for breast cancer and extends the survival of patients with advanced breast cancer. However, chemotherapy-induced IHD poses a serious threat to the long-term survival for patients with BC, which has raised widespread concern among oncologists and cardiologists. Anthracyclines are the most commonly used chemotherapy drugs for BC, but they often exhibit a wide range of cardiotoxicity. Clinically, overt cardiotoxicity is observed in approximately 6% of cases, while subclinical manifestations are detected in around 18% [104]. Notably, cardiovascular mortality represents the primary cause of death among BC survivors who experience heart failure following anthracycline chemotherapy [105]. Anthracycline-induced cardiotoxicity may present as acute (occurring immediately after infusion), early (within the first year of treatment), or late (emerging several years post-treatment). Acute cardiotoxicity, observed in approximately 1% of patients, is characterized by supraventricular arrhythmias, transient left ventricular dysfunction, and electrocardiographic abnormalities [106]. Early-onset cardiotoxicity, the most prevalent form of anthracycline-induced cardiotoxicity, is characterized by left ventricular dysfunction, reduced left ventricular ejection fraction (LVEF), and symptomatic heart failure, occurring in approximately 5% of patients [107]. Late-onset cardiotoxicity, predominantly presenting as cardiomyopathy, is characterized by cytoplasmic vacuolization of cardiomyocytes resulting from sarcoplasmic and mitochondrial swelling, organelle destruction, cardiomyocyte death, and disruption of muscle fiber organization [108]. Coronary artery disease, a form of IHD, has been observed in patients receiving anthracycline therapy, particularly in those undergoing combined treatment with anthracyclines and radiotherapy [109]. Taxanes are indispensable therapeutic agents in the treatment of both early-stage and many advanced-stage BC, such as paclitaxel and docetaxel. However, they can also induce cardiotoxicity, leading to myocardial ischemia, coronary artery thrombosis, and MI [110–113]. In vitro, paclitaxel impaired the proliferation and migration capacity of endothelial cells by affecting microtubule dynamics [114], which could potentially accelerate the progression of atherosclerosis. The capecitabine, as an antimetabolic antitumor drug, significantly reduces the risk of recurrence in postoperative triple-negative breast cancer and continues to play a pivotal role in second-line therapy for many advanced BC. However, like its metabolite 5-fluorouracil, both have potential risks of cardiac damage [115]. The mechanism underlying myocardial ischemia and MI induced by 5-fluorouracil is not fully understood, but coronary artery spasm is believed to be the primary cause. The constriction of coronary artery and branch artery were observed by echocardiography and coronary angiograph [116, 117]. Additionally, Mosseri et al. found that 5-fluorouracil can induce coronary artery vasoconstriction independent of endothelial cell injury via the protein kinase C pathway in animal models [118]. Another mechanism that 5-fluorouracil causes MI maybe its damages to the coronary artery endothelium. On one hand, as a cytotoxic agent, 5-fluorouracil can directly damage vascular endothelial cells and lead to microthrombi, and subsequent myocardial ischemia cannot be blocked by calcium channel blockers [119]; On the other hand, 5-fluorouracil can also increase lipid peroxidation of cell membranes and intracellular oxidative stress mediated by reactive oxygen species, leading to endothelial cell damage and apoptosis. Gemcitabine is also commonly used chemotherapy drug in second-line chemotherapy regimens for BC, playing a crucial role in salvage therapy for BC. Numerous case reports have shown that it can also trigger coronary artery spasm, myocardial ischemia, and MI [120–122]. Therefore, the cardiac toxicity of gemcitabine may be attributed to coronary artery spasm and angina pectoris. Persistent spasm may lead to acute MI, but other mechanisms that may also cause coronary ischemia, such as endothelial dysfunction and intracoronary thrombosis, awaiting confirmation by further researches.

Approximately 80% of patients with BC are hormone receptor-positive [123], the National Comprehensive Cancer Network (NCCN) guidelines recommend that patients with estrogen receptor and/or progesterone receptor-positive tumors should receive endocrine therapy. Tamoxifen have significantly improved the prognosis of patients with hormone receptor-positive BC by binding to the estrogen receptor and inhibiting the growth of BC cells [11]. In postmenopausal patients, the main source of estrogen is the aromatase-mediated conversion of androgens to estrogens, and aromatase inhibitor (AI) can inhibit this process, which contributes a significant reduction in estrogen levels in the body and achieves the therapeutic goal of treating BC [12]. However, the decline in estrogen levels in postmenopausal women is significantly associated with an increasing incidence and mortality of cardiovascular events [124], which may be attributed to the loss of estrogen’s protective effect on the cardiovascular system. Prior research has indicated that estrogen may exert its cardiovascular protective effects through estrogen receptor-mediated rapid or membrane-initiated (non-genomic) signaling cascades to regulate vascular tone [125]. Furthermore, endogenous estrogen can modulate lipid metabolism processes within the body, including regulating cholesterol metabolism, promoting fat oxidation, facilitating lipid transport, and enhancing metabolic activity in adipocytes, which will retard the progression of atherosclerotic plaques [126]. Hence, the utilization of AI may exacerbate myocardial ischemic lesions, thereby increasing susceptibility of MI in patients receiving AI [127]. Tamoxifen competitively binds to estrogen receptors and exerts anticancer effects, which theoretically could also impact estrogen’s protective effects on the cardiovascular system. A cohort study revealed that women who experienced MI were more likely to have received tamoxifen therapy than those without MI (HR = 1.71, 95% CI: 1.38–2.13), with a significantly elevated risk of MI observed in patients receiving tamoxifen for more than 5 years compared to those with shorter treatment durations [128]. On the contrary, several studies have indicated that tamoxifen might reduce the risk of MI [129–131], which has been attributed to the protective effects on the cardiovascular system by tamoxifen’s regulatory effect on lipid metabolism and its estrogen-like action. Therefore, some endocrine therapy regimens may potentially promote the myocardial ischemic lesions, which is need to be confirmed by further researches.

In addition to the antitumor therapies mentioned above, other anticancer agents may also contribute to the development of IHD. Anti-HER2 targeted therapy, a pivotal treatment for HER2-overexpressing breast cancer, is also associated with significant cardiotoxicity. Left ventricular dysfunction and heart failure are relatively common cardiotoxicity among patients with anti-HER2 targeted therapy [106]. Moreover, immunotherapy, such as immune checkpoint inhibitors (ICIs), is widely used in metastatic and neoadjuvant/adjuvant BC treatment, especially in TNBC. While, emerging evidence highlights immune responses within the cardiovascular system, particularly the heart, in patients undergoing ICIs treatment [132]. Myocarditis is a common and fatal side effect of ICIs. Atherosclerosis is also often seen in patients using ICIs, which can cause arterial thrombotic events. A retrospective study showed that 1% of patients had MI or ischemic stroke within 6 months of receiving ICIs [133]. ICIs mediated T cell activation plays an important role in the occurrence and development of atherosclerosis [134]. Thus, IHD caused by immunotherapy should not be ignored in patients with BC.

### The impact of IHD management on BC

IHD poses a significant threat to population health, with MI representing the most acute and highly fatal cardiovascular event [45]. Therefore, the prevention and treatment of IHD are particularly crucial for improving the health and longevity of the whole population. Coronary artery atherosclerosis is the primary cause of myocardial ischemia and may lead to the occurrence of MI. Therefore, preventing the occurrence of coronary artery atherosclerosis and slowing its progression are key measures in treating IHD. Previous research has indicated that unhealthy lifestyle factors, such as lack of physical activity, smoking, and obesity, are associated with an increased risk of developing coronary artery atherosclerosis [135–137]. Additionally, multiple abnormalities within metabolic syndrome also contribute to an increased risk of developing atherosclerosis [138–140]. Therefore, maintaining the stable levels of blood pressure, blood glucose, and blood lipid is also crucial for the prevention and treatment of atherosclerosis. Although many treatment drugs of diabetes have anti-breast cancer effects, certain hypoglycemic agents may also potentially promote the occurrence and progression of BC. Previous studies have consistently demonstrated that insulin glargine will increase the risk of BC [141–143]. Another meta-analysis comprising 42 studies also showed that the risk of BC (RR = 1.14, 95%CI: 1.01–1.29) slightly increased in diabetic patients using glargine insulin compared to the patients using non-glargine insulin [144]. Nevertheless, the carcinogenic mechanisms of glargine insulin remain incompletely elucidated. Some studies have indicated that the exposure of breast epithelial cells to insulin may promote the stepwise carcinogenic transformation process [145, 146], which may be attributed to the inhibited apoptosis of transformed breast epithelial cells by the activated members of the insulin-like growth factor receptor family. Therefore, appropriate hypoglycemic agents should be considered to reduce the risk of BC when slowing the progression of coronary artery atherosclerosis by controlling blood glucose. In addition to adopting healthy lifestyle practices and controlling metabolic syndrome, some antiplatelet aggregation drugs are also commonly employed in the primary prevention of MI. Activated platelets will induce the secretion of chemotactic factors from endothelial cells and smooth muscle cells, such as monocyte chemotactic protein-1 (MCP-1) and Interleukin-8 (IL), which will exacerbate endothelial cell injury and inflammatory response within the vasculature by recruitment and extravasation of leukocyte [147]. Furthermore, previous experimental and clinical studies have shown that platelet can promote the initiation of atherosclerosis during the early-stage disease (vascular inflammatory phase) and contribute to the long-term progression of atherosclerotic thrombosis [148, 149]. Platelet can adhere to exposed subendothelial collagen and von Willebrand factor (vWF) within the vasculature when the rupture of atherosclerotic plaque occurs [150–152], which will trigger coronary artery thrombosis and myocardial ischemic injury. Uncontrolled accumulation of platelet around the ischemic myocardium will impair microcirculatory function and promote myocardial dysfunction [153, 154]. Therefore, antiplatelet therapy is crucial for the prevention and treatment of cardiovascular diseases. Currently, long-term antiplatelet therapy strategies primarily include single antiplatelet therapy (SAPT) and dual antiplatelet therapy (DAPT). DAPT is the standard regimen for antiplatelet therapy in patients with chronic coronary syndrome following percutaneous coronary intervention (PCI). Although antiplatelet therapy is paramount in the prevention and treatment of cardiovascular diseases, two previous studies have demonstrated that prolonged use of clopidogrel is associated with an increased risk of cancer-related mortality [155]. In an animal experiment, treatment with DAPT in 4T1 breast tumor-bearing mice contributed to the tumor progression and shortened survival of mice [156]. In another cellular experiment, co-treatment with anastrozole and DAPT was found to promote the survival of MCF7 and T47D cells and induce more invasive breast cancer phenotypes [157]. Further investigation suggests that under the conditions of anastrozole and DAPT treatment, MCF7 and T47D cells may utilize the initial cytotoxic effect as a selective pressure through the autophagy pathway to promote tumor cell survival and metastasis [158]. Therefore, antiplatelet therapy should also be approached with caution in patients with BC.

MI is the most ominous condition within IHD, with an exceedingly high fatality rate [44, 45]. Once MI occurs, particularly in the case of ST-segment elevation MI, prompt reperfusion therapy is crucial for reducing mortality and complications. Primary percutaneous coronary intervention (PCI) is the preferred reperfusion therapy when the time from first medical contact (FMC) to wire passage through the infarct-related artery (IRA) is <120 min. However, in settings without access to PCI or in cases where the anticipated time from FMC to wire passage through the IRA exceeds 120 minutes, initiating thrombolytic therapy within 10 minutes of FMC is also a feasible option. Thrombolytic drugs mainly include urokinase, recombinant human tissue-type plasminogen activator, alteplase, and etc. They directly or indirectly activate plasminogen and convert it into plasmin, which will degrade fibrinogen in thrombi and dissolve thrombi, ultimately restoring myocardial perfusion by reopening obstructed coronary arteries. Studies have shown that plasmin activated by urokinase-type plasminogen activator (uPA) can also degrade extracellular matrix (ECM) components, which will facilitate tumor cells invasion through basement membrane and developing distant metastasis [159–161]. Therefore, in patients with concomitant BC and MI undergoing thrombolytic therapy, activated plasmin may promote distant metastasis of breast cancer cells. Furthermore, previous studies have demonstrated higher expression of urokinase-type plasminogen activator receptor (uPAR) in breast cancer, particularly in TNBC [162, 163], the binding of uPA to uPAR facilitates cancer cell proliferation, survival, migration, and invasion [164, 165]. The latest study demonstrates that the combination of sodium 4-phenylbutyrate and mouse urokinase amino-terminal fragment targeting the overexpressed uPAR on the surface of TNBC cells significantly inhibited the proliferation and metastasis of TNBC cells [166]. Therefore, theoretically, during thrombolytic therapy using uPA, high concentrations of uPA in vivo may also activate uPAR-related signaling pathways to promote distant metastasis of BC cells.

In addition to reperfusion therapy, other pharmacological treatments play a crucial role in IHD. Anticoagulant and antiplatelet therapy is not only the cornerstone of pre-thrombolysis treatment but also an essential component of post-thrombolysis therapy for MI. However, many treatment regimens for BC can lead to bone marrow suppression characterized by thrombocytopenia, including platinum-based chemotherapy drugs, CDK4/6 inhibitors, and trastuzumab-emtansine, and etc [167–169]. In patients with severe thrombocytopenia, anticoagulant therapy carries a higher risk of bleeding. Therefore, in breast cancer patients with concomitant MI, anticoagulant therapy should take into account the patient’s coagulation function to reduce the occurrence of bleeding-related complications. Statins are widely used in the management of IHD due to their lipid-lowering and anti-inflammatory properties. They inhibit HMG-CoA reductase, reducing LDL cholesterol and stabilizing atherosclerotic plaques [170]. Statins can also improve endothelial function, decrease oxidative stress, and reduce systemic inflammation. These effects help prevent acute coronary events, such as MI, and improve long-term cardiovascular outcomes. However, cells treated with statins demonstrated increased migratory capacity, characterized by significantly higher velocity and greater accumulated distance, alongside the upregulation of migration-associated genes [171]. Thus, statins may increase the risk of BC metastasis. Vitamin B3, as a vitamin-class medication, can exert vasodilatory effects and alleviate vascular spasms to improve local blood supply, and it can exert favorable therapeutic effects in ischemic heart disease, such as MI and angina pectoris. However, recent studies have shown that nicotinamide, derived from vitamin B3 in the body, supplementation of which in excess may increase the incidence of tumors and the risk of TNBC brain metastasis [172]. Therefore, caution should be exercised in the use of vitamin B3 in the treatment of BC complicated by MI.

## Discussion and recommendations

Although BC and IHD originate from different physiological systems, they are not entirely independent diseases and exert mutual influence on each other. The occurrence of breast cancer may potentially trigger IHD and accelerate its progression. As mentioned above, various pro-inflammatory cytokines produced in the tumor microenvironment will damage the heart vessels and cause IHD, such as TNF-α, IL-1β, IL-6 and etc [55, 65, 72]. By contrast, IHD, especially MI, also exerts a promotive effect on the occurrence and exacerbation of BC. After MI, cardiac interstitial stromal cells release extracellular vesicles enriched with specific biomolecules, such as osteonectin, IL-6, galectin-3, TNF-α, and VEGF, which exhibit reparative effects on the heart. However, these biomolecules may also contribute to the progression of BC [91]. Another study conducted by New York University indicated that MI leads to immune cell reprogramming, driving certain immune cells to adopt an immunosuppressive phenotype. This process results in systemic immune dysregulation, thereby accelerating the progression and metastasis of BC [92]. Additionally, their treatments may also exert mutual influence. Many anticancer treatments for BC carry a risk of cardiovascular damage, especially IHD and MI. Conversely, in the treatment regimens for IHD, there are also risks of promoting tumorigenesis and exacerbating tumor progression, which pose potential risks to the occurrence and development of BC. Therefore, considerations about their potential interplay mechanisms are necessary to minimize their mutual impact as much as possible in treating these two diseases.

### Reducing the risk of IHD in BC patients

Although breast cancer ranks first in the incidence of malignant tumors among women [1], the long-term survival of patients with BC has been significantly improved due to relatively comprehensive anticancer treatment regimens, with 5-year survival rate of 93.6% [22]. A cohort analysis based on breast cancer survivors revealed that cardiovascular disease becomes the leading cause of death among patients with BC as the follow-up time extends [27]. Therefore, greater attention should be paid to the prevention and treatment of associated cardiovascular diseases to improve the long-term survival for patients with BC, such as IHD relating to BC.

#### Prevention of IHD through healthy lifestyles

Coronary artery stenosis and MI resulting from coronary atherosclerosis are the primary types of IHD. Therefore, patients with BC should pay more attention to the prevention of atherosclerosis, and maintaining a healthy lifestyle is an essential component of atherosclerosis prevention. Previous studies have shown that exercise can significantly slow the progression of atherosclerosis [135, 173], and appropriate physical activity can also reduce the risk of breast cancer-related mortality [174]. Therefore, breast cancer patients should develop a rational exercise plan that does not interfere with anticancer treatments. In addition to affecting respiratory system diseases, smoking quantity is positively correlated with the severity of atherosclerosis and significantly increases the risk of atherosclerosis-related mortality [136]. Therefore, smoking cessation is also an indispensable component for some patients with BC in preventing atherosclerosis. Obesity is not only a risk factor for BC [175], but also a significant contributor to atherosclerosis [176]. Therefore, patients with BC should prioritize weight management. Furthermore, lipid abnormalities also play a crucial role in the progression of atherosclerosis [31, 177]. Many treatment regimens for BC can also lead to lipid abnormalities, such as certain chemotherapy and endocrine therapies. Thus, patients with BC should enhance the monitor of blood lipid level and undergo lipid-lowering therapy if necessary.

#### Optimizing breast cancer treatment strategies

As the survival duration of patients with BC extends, treatment-related cardiotoxicity becomes increasingly evident. Anticancer treatments should also strive to avoid schemes causing myocardial ischemic damage as much as possible. It is advisable to conduct a comprehensive evaluation of cardiac function through electrocardiography, echocardiography examination, and cardiac injury markers before adjuvant therapy. The potential risk of cardiac damage from radiotherapy remains significant, and efforts should be made to minimize radiation-related cardiac injury as much as possible. Some emerging radiotherapy techniques, such as intensity-modulated radiotherapy (IMRT), improve dose uniformity in the target area, which will enhance the efficacy and reduce cardiac and normal tissue injury [178, 179]. In cases with large target curvature and thin chest walls, volumetric-modulated arc therapy (VMAT) demonstrates more pronounced cardiac sparing compared to IMRT [180]. During radiotherapy, adopting the deep inspiration breath-hold (DIBH) technique is beneficial for reducing cardiac irradiation dose. And studies have shown that DIBH-VMAT further decreases the dose delivered to the heart [181]. During anticancer treatment, it is essential to monitor patients’ electrocardiographic changes and symptoms related to IHD. Prompt intervention should be provided in the event of IHD. Simultaneously, researchers are encouraged to delve into the mechanisms about the IHD induced by chemotherapy and other drugs to develop related cardioprotective medications. As mentioned above, various pro-inflammatory cytokines associated with breast cancer, such as TNF-α [55, 56], IL-1β [64, 65], and IL-6 [71, 72], may also trigger and exacerbate coronary atherosclerosis, increasing the risk of MI. Therefore, concurrent use of anti-inflammatory agents during breast cancer treatment may have certain effects on delaying the progression of atherosclerosis. For example, previous studies have shown that targeting IL-1β with canakinumab significantly reduced the recurrence rate of cardiovascular events in patients with MI [182]. Tocilizumab is a monoclonal antibody that binds to both membrane-bound and soluble IL-6 receptors, improving endothelial function, reducing oxidative stress, and attenuating the thrombotic and inflammatory properties of monocytes [183]. Furthermore, another IL-6 inhibitor, Ziltivekimab, significantly reduced biomarkers of inflammation and thrombosis associated with atherosclerosis [73]. Therefore, the use of these agents targeting cytokines in patients with BC may help reduce the incidence of IHD.

### Reducing the risk of BC in patients with MI

As mentioned above, the risk of developing BC significantly increases in patients after MI [84, 85]. Therefore, elucidating the mechanism about the effect of myocardial infarction on the occurrence of BC can help reduce the risk of cancer in patients with MI. Some scholars attributed the higher risk of BC after MI to their shared risk factors, such as obesity, smoking, and lack of physical activity [86–90, 184]. Therefore, patients with a history of MI should develop a reasonable exercise plan and control their weight after the condition stabilizes. Also, patients with a history of smoking should be advised to quit smoking. Moreover, due to the synergistic effect of common risk factors in the early stages of both conditions, increasing breast examinations during follow-up of MI can facilitate the early detection of BC. Additionally, female patients are more prone to experience anxiety and depression following MI [185], which also significantly increases the incidence of malignant tumors [186]. Therefore, addressing maladaptive psychological states in post-MI patients may also contribute to reducing the incidence of BC.

### Treatment approaches for IHD complicated by BC

#### Treatment of acute MI complicated by BC

BC and IHD mutually influence and exacerbate each other, which poses significant threats to patients’ physical health and life safety. Collaboration between oncologists and cardiologists is often necessary to develop individualized treatment plans when IHD coexists with BC, which also promotes the advancement of cardio-oncology. Acute MI represents the most critical condition within IHD, with extremely high rates of disability and mortality. Only timely and effective treatment can restore myocardial reperfusion and reduce the total ischemic time and the area of necrotic myocardium. Therefore, when acute MI is complicated by BC, the focus should primarily be on the treatment of MI. According to the European Society of Cardiology guidelines [187], it is crucial to strive for completing PCI treatment within 120 minutes of initial medical contact for ST-segment elevation MI, as this is essential for reducing complications and mortality rates. When it is not feasible to open the occluded coronary artery causing MI through PCI within 120 minutes, thrombolytic therapy should be administered as soon as possible (within 10 minutes). However, the activated plasminogen activator might also promote the distant metastasis of breast cancer cells by degrading the extracellular matrix [159–161]. Moreover, the signaling pathway activated by the binding of uPA to uPAR facilitates cancer cell proliferation, survival, migration, and invasion [164, 165]. In addition to the above treatments, anticoagulant and antiplatelet therapy are also crucial in the treatment of acute MI. Anticoagulant drugs, such as low molecular weight heparin, significantly improve the prognosis for patients with acute MI [188]. However, anticoagulant therapy requires close monitoring the changes in coagulation function to reduce the risk of bleeding in breast cancer patients.

#### Treatment of BC combined with other IHD

In addition to acute MI, IHD, such as coronary artery atherosclerosis and its relating stable and unstable angina, pose significant potential threats to cancer patients, as well. Therefore, patients with BC who have symptoms of coronary artery disease and high risks of MI should undergo comprehensive coronary artery imaging examinations to assess the degree of coronary artery stenosis, such as coronary CT angiography and coronary angiography. The optimal diagnosis and treatment plan can be devised under the multidisciplinary treatment of cardiologists, oncologists, and anesthesiologists. In patients with mild coronary artery stenosis caused by coronary atherosclerosis and stable angina, concurrent with anticancer therapy, medicine can be pursued to alleviate myocardial ischemic symptoms and reduce the risk of myocardial infarction, such as lipid-lowering therapy, antiplatelet therapy, relief of coronary spasm, and reduction of myocardial oxygen consumption. For patients with severe coronary artery stenosis ( ≥ 75%) and unstable angina, in addition to basic medicine therapy, interventional procedures and coronary artery bypass graft surgery may be preferable options. Furthermore, the management of breast cancer-related treatments should aim to minimize exacerbation of ischemic cardiac conditions. Close monitoring of ischemic cardiac-related symptoms and pertinent laboratory and imaging changes should be conducted during anti-tumor therapy.

### Potential drugs targeting both IHD and BC

Although certain treatments for IHD and BC may interact with each other, some potential drugs could target both conditions simultaneously. Sodium-glucose co-transporter 2 (SGLT2) inhibitors are primarily used in the treatment of diabetes, but emerging evidence also suggests they exhibit beneficial effects in both tumor management and cardiovascular disease. A basic study showed that the SGLT2 inhibitors, dagaglizin and caglizin, have a powerful anti-proliferative effect on BC cells by blocking cell cycle and inducing cell apoptosis [189]. In terms of cardiovascular disease, SGLT2 inhibitors also exhibited a good therapeutic effect. A meta-analysis demonstrated that SGLT2 inhibitors were associated with a 13% reduction in the risk of mortality among patients with atherosclerotic cardiovascular disease (HR 0.87, 95% CI 0.78–0.97) [190]. Everolimus combined with exemestane has shown promising effect in hormone receptor-positive, human epidermal growth factor receptor-2 negative BC [191]. Everolimus is also utilized in the treatment of cardiovascular diseases, with Everolimus-Eluting Stents (EES) demonstrating efficacy in preventing restenosis and thrombosis following stent implantation through the synergistic action of the drug and stent [192]. β-receptor blockers, widely used in the management of cardiovascular diseases, are effective in reducing the risk of recurrent MI and alleviating angina in the short term [193]. Interestingly, it can also inhibit BC cell proliferation by disrupting cell cycle progression and cyclin homeostasis levels [194]. Therefore, the aforementioned drugs may represent promising therapeutic options due to their dual benefits in managing both IHD and BC.

## Summary and future directions

BC and IHD are not entirely independent conditions, they exert mutual influences on each other. The occurrence of BC can trigger and accelerate the progression of coronary artery atherosclerosis, ultimately leading to IHD and increasing the risk of mortality in breast cancer patients. Conversely, ischemic heart disease may potentially have a promoting effect on the occurrence of breast cancer and accelerate the progression of breast cancer. However, most of these findings are derived from retrospective analyses, and the specific mechanisms underlying the interaction between IHD and BC are still not fully elucidated. Therefore, more rigorous clinical and basic researches are needed to further elucidate the interaction and specific mechanisms between BC and IHD, which will contribute to improving the long-term survival for patients with BC and reducing the incidence risk of tumors in patients with IHD.

In addition to the diseases themselves, the treatment of BC and IHD can also exert mutual influences. Many BC treatments will promote the occurrence and progression of IHD, such as radiation therapy, chemotherapy, and endocrine therapy. Management of IHD can also accelerate the progression of BC, such as thrombolysis and antiplatelet treatments. Therefore, in-depth investigation into the specific mechanisms underlying the interaction between anticancer therapy and IHD management will facilitate the optimization of therapeutic strategies, thereby improving the prognosis for patients with BC and/or IHD.
